# Single-cell sorting based on secreted products for functionally defined cell therapies

**DOI:** 10.1038/s41378-022-00422-x

**Published:** 2022-07-22

**Authors:** Hiromi Miwa, Robert Dimatteo, Joseph de Rutte, Rajesh Ghosh, Dino Di Carlo

**Affiliations:** 1 Department of Bioengineering, University of California - Los Angeles, Los Angeles, CA 90095 USA; 2 Department of Chemical and Biomolecular Engineering, University of California - Los Angeles, Los Angeles, CA 90095 USA; 3 Partillion Bioscience, Los Angeles, CA 90095 USA; 4 Department of Mechanical and Aerospace Engineering, University of California - Los Angeles, Los Angeles, CA 90095 USA; 5 California NanoSystems Institute (CNSI), University of California - Los Angeles, Los Angeles, CA 90095 USA

**Keywords:** Microfluidics, Nanoscience and technology

## Abstract

Cell therapies have emerged as a promising new class of “living” therapeutics over the last decade and have been particularly successful for treating hematological malignancies. Increasingly, cellular therapeutics are being developed with the aim of treating almost any disease, from solid tumors and autoimmune disorders to fibrosis, neurodegenerative disorders and even aging itself. However, their therapeutic potential has remained limited due to the fundamental differences in how molecular and cellular therapies function. While the structure of a molecular therapeutic is directly linked to biological function, cells with the same genetic blueprint can have vastly different functional properties (e.g., secretion, proliferation, cell killing, migration). Although there exists a vast array of analytical and preparative separation approaches for molecules, the functional differences among cells are exacerbated by a lack of functional potency-based sorting approaches. In this context, we describe the need for next-generation single-cell profiling microtechnologies that allow the direct evaluation and sorting of single cells based on functional properties, with a focus on secreted molecules, which are critical for the in vivo efficacy of current cell therapies. We first define three critical processes for single-cell secretion-based profiling technology: (1) partitioning individual cells into uniform compartments; (2) accumulating secretions and labeling via reporter molecules; and (3) measuring the signal associated with the reporter and, if sorting, triggering a sorting event based on these reporter signals. We summarize recent academic and commercial technologies for functional single-cell analysis in addition to sorting and industrial applications of these technologies. These approaches fall into three categories: microchamber, microfluidic droplet, and lab-on-a-particle technologies. Finally, we outline a number of unmet needs in terms of the discovery, design and manufacturing of cellular therapeutics and how the next generation of single-cell functional screening technologies could allow the realization of robust cellular therapeutics for all patients.

## Graphical abstract

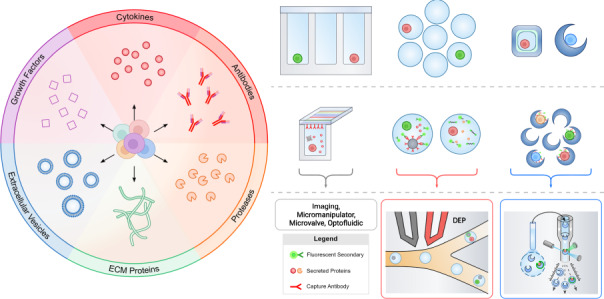

## Introduction

Decades of advancement in genetic engineering, bioprocessing, and basic medical science have fostered the emergence of cellular therapeutics as a novel pillar of medicine^1^. These approaches transform populations of cells into armies of living drugs that can proliferate within a patient and provide long-term treatments for otherwise intractable chronic and systemic illness. However, as the field continues to progress, it is becoming clear that we are still fundamentally limited in our understanding of the phenotypic traits that endow cells with therapeutically beneficial properties^2–6^ and how to engineer and select cells with these traits^7^.

**Fig. 1:**
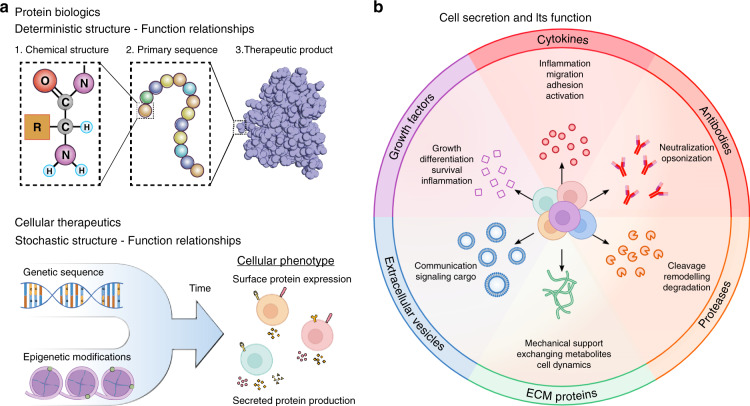
Contrasting protein and cellular therapeutics with a focus on cell secretory function. **a** Molecular therapeutics, such as protein biologics, are defined by their molecular structure, such as amino acid sequence. Cellular phenotype is controlled by input not only from the genome but also from the relative silencing and activity of specific genes, which are largely controlled by epigenetic modifications. Furthermore, the spatial localization and phosphorylation state of intracellular biomolecules, also play key roles, which change with time and external environmental inputs. Therefore, cell function is a stochastic process only loosely linked to structural features within the cell. **b** Cells secrete numerous factors, including proteins and extracellular vesicles, into their surroundings to shape their local microenvironment. Families of proteins such as growth factors and cytokines signal to other types of cells to coordinate tissue regeneration and differentiation or to coordinate immunological responses to infection. Specialized molecules known as antibodies are produced by B cells and serve to inhibit pathogen function while simultaneously alerting the immune system to their presence. Proteases such as matrix metalloproteinases (MMPs) degrade extracellular matrix (ECM) proteins to breakdown tissues, and new ECM proteins can also be secreted as a scaffold for cell growth after injury. Extracellular vesicles such as exosomes are small vesicles that deliver intracellular proteins, RNAs, and DNAs directly to the cytoplasm of recipient cells, altering their behavior. The development of novel systems to study the types and relative amounts of products secreted by individual cells will yield tremendous benefits for advancing basic biological understanding and novel cellular therapeutic applications.

Cells can execute complex tasks that cannot be achieved with small-molecule drugs or biologics. In optimal scenarios, populations of therapeutic cells can home to sites of disease, integrate environmental cues to modulate the intensity of their response (which can include secreting communication factors, enzymes or cytotoxic compounds), and survive and proliferate to prevent disease relapse. Unfortunately, the complexity of these behaviors also renders the design and characterization of such drugs immensely more difficult^8–10^. Unlike small molecules or proteins, the functional potency of cells cannot be ascertained from simple metrics such as primary structure or affinity. Mutations at both the genetic^11^ and epigenetic^12^ levels may confer functional advantages on select cell clones, which translate to variations in biological potency in vivo (Fig. 1). Similarly, differences in expansion protocols may result in the exhaustion or selective proliferation of subsets of cells.

The strong linkage between the structure and function of molecular therapies cannot be relied upon for cell therapies, requiring new methods to measure and quantify cell therapy function^7^. Molecular therapies, such as protein biologics, are defined by their molecular structure, such as amino acid sequence, and therefore methods to measure structure can be used to confirm the function of the therapeutic (Fig. 1a)^13^. Tools such as chromatography and electrophoresis are used to measure and separate proteins based on size and shape^14^, and mass spectrometry can uncover the amino acid sequence^15^. Affinity binding measurements can be directly linked to proper protein shape and function^16,17^. Ultimately, there is a deterministic link between the correct protein structure and its potency as a therapeutic. This linkage is largely lost or extremely difficult to measure for cell therapies. The structure of the cell has inputs from the genome but also from the relative silencing or activity of specific genes, which is largely controlled by epigenetic modifications^18^. The time history, spatial localization of molecules, and phosphorylation states of proteins can also play a role. Although some aspects, such as genetic underpinnings or the mRNA transcripts produced by individual cells, can be measured destructively^19^, the ability to use this information to predict a specific cell’s function is still largely out of reach. Therefore, cell function is a stochastic process loosely linked to structural features within cells (Fig. 1b), and processes to introduce or separate cells based on structural features cannot alone be used to define a functional cell therapy product. As an example, the presence of a chimeric antigen receptor (CAR) protein in the membrane of a CAR T-cell does not provide sufficient information to know the potency of the cell, taking into account the potential for T-cell exhaustion, metabolic dysfunction, or signaling abnormalities^20^. Further advancements to design and standardize cellular therapies will rely on foundational technologies that can (1) rapidly and reliably probe cellular function to identify the quality metrics critical for therapeutic design and (2) enrich for cells with desirable functional properties to standardize optimal therapeutic formulations.

Perhaps the most direct metric of functional potency can be found by measuring the propensity of cells to secrete bioactive proteins (Fig. 1c). Indeed, the vastness of the secretome underscores its importance in a wide array of biological processes. Of the ~20,000 protein coding genes, ~3000 proteins (15%) are predicted to be secreted, compared to ~5000 membrane-bound proteins^21^. These secreted factors lead to outsized biological effects compared to membrane-bound molecules because of their ability to diffuse and rapidly remodel signaling between many nearby cells as well as travel systemically through the circulation. Local increases in growth factor concentrations can aid the regeneration and maturation of vasculature, enabling tissue regrowth after traumatic injury^22^. Cytokines and chemokines work in concert to coordinate the activity of immune cells as they respond to pathogens or mutated cells^23^. Extracellular matrix (ECM) components are secreted and structured to provide specialized scaffolding for various tissues^24^. Proteolytic enzymes catalyze reactions in the cellular microenvironment, proving critical in functions such as ECM remodeling during inflammation and wound healing^25^. Extracellular vesicles are a newly discovered mode of intercellular communication. Interactions of extracellular vesicles (including exosomes and microvesicles) with recipient cells can have various effects on the target cell, from stimulating signaling pathways to providing trophic support, depending on the mode of interaction and on the intracellular fate of the vesicles in the case of their uptake^26^. Finally, antibodies have multiple crucial roles in the humoral response, including neutralizing pathogens by blocking surface antigens and marking pathogens for destruction by phagocytic cells, such as macrophages or neutrophils^27^.

Unfortunately, the functional profiling and selection of cell populations as a base for cellular therapeutics is not standard in therapeutic pipelines^28^. Furthermore, in the small fraction of cases where profiling approaches are carried out, researchers focus on selection via differentially expressed surface molecules, factors that allude to cellular role but are not fully descriptive of inherent bioactivity^29^. There is still a critical need for techniques that can augment current selection approaches with this complementary set of secretory data. Recently, numerous proof-of-principle approaches have been reported in the literature but are not yet broadly available. The continued design and optimization of such phenotyping technologies, particularly platforms that profile individual cell clones massively in parallel, will become invaluable design tools for the engineering of more complex next-generation cell therapies.

From this perspective, we highlight the state of the art in single-cell secretion-based profiling technology, with a special emphasis on its utility in cell therapy development and the factors that limit its widespread adoption. We begin by reviewing the key engineering principles leveraged by researchers in the design of such platforms as a basis for understanding the underlying theory and inherent design constraints of these systems. Next, we review the key emerging technologies and highlight their current applications while simultaneously comparing the strengths and shortcomings of each approach. Finally, we conclude with a perspective on how these technologies can be further engineered to better address unmet needs in the development of next-generation immune and stem cell therapeutics. We hope that this work will foster collaboration between scientists designing cell therapies and engineers developing tools to refine the design and selection of cells based on their function. We envision that automated functional selection tools will be an essential component in creating future cellular therapeutics.

## Single-cell secretion-based screening concepts

**Fig. 2:**
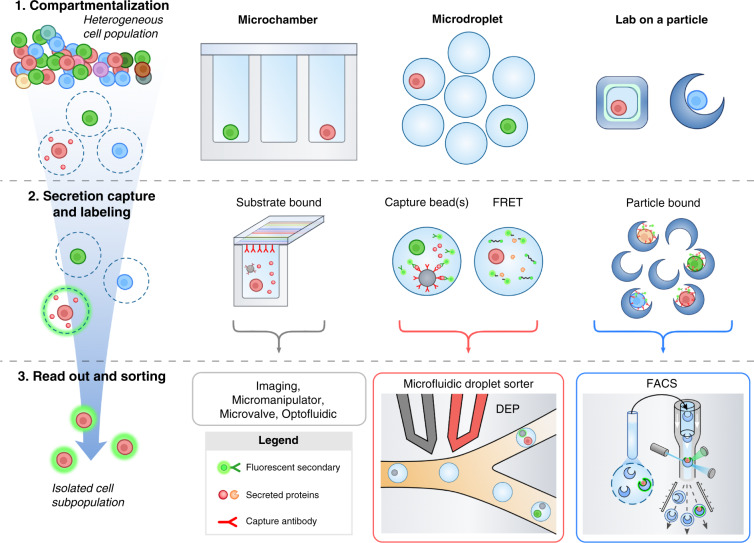
Three Fundamental Steps for Single-Cell Secretion Screening. The analysis of secreted products from individual cells relies on three fundamental processes. **1** Cells are compartmentalized into individual containers so that their secreted products are concentrated. In these compartments, the transport of secretions to neighboring cells, which leads to crosstalk, is reduced. This can be accomplished using microfabricated compartments, aqueous emulsions, or particle-based confinement approaches. **2** Secreted molecules accumulate and are converted into detectable signals. One common method to accumulate and label secretions is to use a sandwich of affinity capture elements such as antibodies. Capture antibodies concentrate secretions onto a solid phase, such as glass slides or capture beads, where they can be detected with secondary fluorescently labeled reporter antibodies. For bioactive molecules, such as enzymes, enzyme substrates that fluoresce upon cleavage enable detection by separating the quencher from the fluorescence donor in a fluorescence resonance energy transfer (FRET) system. **3** Finally, to recover cells of interest, each compartment must be assessed and potentially sorted based on the intensity of the accumulated secretion signal. To recover cells from microwells, low-throughput liquid handlers, microvalves, optofluidics and micromanipulators are needed. Droplets are typically sorted directly on microfluidic chips using techniques such as dielectrophoresis (DEP), in which spatially varying electric fields generated by electrodes (gray and red structures) exert forces on polarizable droplets. Lab-on-a-particle technologies compatible with commercially available high throughput flow sorters have been demonstrated.

Single-cell secretion assays rely on three critical processes: (1) the partitioning of individual cells into uniform compartments, (2) the accumulation of secretions and labeling via reporter molecules, and (3) the measurement of the signal associated with the reporter and, if sorting, the triggering of a sorting event based on these reporter signals. In this section, we provide background for the fundamental concepts and design considerations used to achieve each of these functions (Fig. 2).

## Compartmentalization

The partitioning of cells into uniform compartments serves several fundamental purposes in the design of single-cell secretion profiling systems. First, the physical segregation of cells reduces intercellular transport, limiting crosstalk between neighboring cell clones and ensuring that the readouts generated in each compartment represent the properties of the cells contained within. The prevention of crosstalk can increase the purity of the functional cells that are identified, improving the eventual purity of a cell therapy product. Second, the localization of cells within small-volume (often nano- or picoliter scale) containers allows the rapid concentration of secreted products, shortening the incubation time required to generate detectable levels of molecules. A shorter incubation time in a confined volume without medium refreshment is more likely to maintain high viability in downstream functional sorting and regrowth applications. Concentrating secreted signals can also allow the identification of cells that secrete products at lower levels, enabling expanded assays beyond the analysis of secreted products with high levels. Last, maintaining uniformity in both the size and shape of the formed compartments normalizes assay conditions between cells, enabling direct comparisons of generated signals across populations. Although there is no ideal compartmentalization strategy for all scenarios, factors such as the desired number of cells to be screened, the average secretion rate, and whether cell recovery is needed can help dictate the type and size of compartments used.

The two most prevalent compartmentalization strategies are physical confinement, using compartments such as microfabricated wells^30,31^ and valved channels^32,33^, or the formation of aqueous droplets within an immiscible oil solution^34–36^. Microfabricated geometries are beneficial in that each compartment is coupled with a solid phase, such as a well plate bottom, rendering them compatible with both adherent and suspended cell types. For adherent cells, this can result in higher viability for downstream assays^37^. Single cells are typically manually seeded into wells or chambers, which results in a random number of loaded cells that follows a Poisson distribution^38,39^. This usually limits the fraction of chambers containing single cells to 10%. However, if the compartments are similar in size to individual cells, they can prevent more cells from being loaded and lead to higher filling fractions^40^. Additionally, microfabricated compartments can be made with removable lids^41^ or coupled with pumps and valves^42–44^ to perform washing steps and deliver reagents to cells after compartmentalization^45^. In contrast, the addition of reagents into stably formed water-in-oil emulsions is nontrivial, limiting most reactions carried out in aqueous droplets to a one-pot format in which all necessary reagents and cells are premixed and encapsulated simultaneously. As a result, for use in droplet-based systems, standard assay formats such as immunoassays that rely on multiple exchange and washing steps must be redesigned to be compatible with a one-pot format. In addition, with limited exceptions, the number of cells encapsulated per droplet in these systems is dictated by Poisson statistics, reducing the proportion of droplets that contain the desired number of cells and thus the effective rate at which single cells can be encapsulated^46^. However, a major advantage of microdroplets is that they can be formed rapidly and continuously, with the final number of compartments limited only by the precursor liquid volume and formation time instead of the footprint of a physical mold, which is not easily scaled. Droplets are also easily stored and can be precisely manipulated using microfluidic systems.

Recently, several groups have demonstrated a lab-on-a-particle-based compartmentalization system in which shaped polymeric microparticles can be used as suspendable microwells, where the presence of a cavity in the microparticle limits convective and diffusive transport^47–49^. Uniform aqueous droplets can also be formed around particles with a surrounding oil phase through simple mechanical agitation^50–53^, further reducing transport. These techniques preserve many of the best facets of both microfabricated wells and droplet-based approaches by enabling the attachment of adherent cells, washing steps, and reagent exchange while retaining the ability to manipulate and sort the formed compartments at high rates using standard flow cytometers and fluorescence-activated cell sorters. The loading of cells within these particles also generally follows Poisson statistics; however, by tuning the cavity size in the particle to the cell diameter, improvements above Poisson loading can be achieved, yielding higher numbers of single-cell events^44^. A particularly interesting study by Destgeer et al. demonstrated that pooled populations of particles with slightly varied geometries can be used for multiplex droplet screens^47^. For example, the morphology of a particle cavity can be altered to serve as a visual barcode denoting the analyte a droplet is measuring, and the cavity size can be tuned to manipulate the dynamic range and limit of detection of the assay.

## Secretion capture and labeling

Once cells are confined, the accumulation of secreted products must be transduced into a detectable signal to enable the qualitative or quantitative assessment of secreted products. The most common approach is the use of sandwich immunoassays. Here, solid surfaces functionalized with secreted analyte-specific antibodies are utilized to concentrate analyte from the surrounding solution, enabling localized detection with fluorescently or enzymatically labeled reporter antibodies. In both microfabricated and lab-on-a-particle-based compartmentalization schemes, capture antibodies can be conjugated directly to the surface of the chamber or particle, enabling signal generation from any subvolume containing a cell^53–55^. In contrast, most droplet-based technologies require secondary solid capture phases, often in the form of antibody-coated polymeric microparticles, to be coencapsulated alongside individual cells^56,57^. Recently, bifunctional antibodies that target both a cell surface protein (e.g., CD45) and a cytokine, available from Miltenyi Biotec, have been employed to capture secreted IL-2 on the cell surface for cells loaded within droplets. This approach avoids the need for an additional solid phase^58^, as the secretion is encoded on the cell itself, which can then be analyzed downstream. Notably, to preserve the fidelity of single-cell assays, it is often desirable to restrict the number of compartments containing more than one cell. This is typically done by diluting the input cell population such that most generated droplets are empty and a small subset of droplets (~10–30%) contain a cell. Such loading inefficiencies grow exponentially in detection schemes relying on the coencapsulation of both a single cell and a single capture phase and can significantly reduce the fraction of an analyzed cell population that can be assayed^59^, which can seriously limit the preparation of large populations for cell therapy manufacturing-based applications^46^. While in principle it is feasible to circumvent some of these issues by loading capture particles in excess such that each droplet will contain a multitude of particles, in practice, the resolution of multiparticle platforms typically suffers because the signals are diffusely scattered throughout each compartment and can be masked by background fluorescence. An interesting workflow reported by Chokkalingam et al. was able to circumvent these limitations by forming droplets in an agarose-rich aqueous phase. This enabled the gelation of droplets after formation, allowing the researchers to wash away background signals from the sample while retaining the secreting cell and capturing beads within the gel mesh^60^. For cell therapy applications, the gel matrix would need to be degraded for eventual therapeutic use. The accumulation of magnetic particles at a specific location in a droplet can also be used to partially overcome the background fluorescence problem in droplets^61^.

Alternatives to fluorescent sandwich immunoassays have also been reported and may be preferred depending on the type of analyte being studied and the limit of detection needed. For example, fluorescence resonance energy transfer (FRET) can be used to characterize affinity reactions or blocking reactions in a solution phase, although the signal-to-noise ratio is less than that in standard washing-based immunoassays^35^. Secreted enzymes with proteolytic activity (such as MMPs) can also be measured using FRET approaches, where initially quenched fluorescent peptide substrates are cleaved by secreted enzymes to generate signals^60,62–64^. Enzymatic FRET analysis typically offers lower limits of detection than fluorescent sandwich immunoassays, as individual enzymes can cleave multiple substrate molecules before losing activity, amplifying the generated signals. However, this phenomenon can also easily lead to increases in the assay false-positive rate, as even a few molecules encapsulated in a compartment away from the secreting cell can generate detectable levels of fluorescence signal. Carefully designed microfluidic systems can be used to wash samples immediately prior to analysis, either passively or actively^64–66^.

Finally, it should be mentioned that while variations of the aforementioned secretion detection approaches exist, the use of antibodies to detect secreted proteins is a consistent feature across the majority of assays reported to date. Selection of the proper antibody or pairs of antibodies is imperative to proper assay performance. Low-affinity (high dissociation constant, K_D_) antibodies can result in false negatives or low signals^36^. Polyclonal antibody mixtures can improve signals by binding to multiple epitopes on the target antigen but also display higher rates of off-target binding. Practically, when designing a secretion assay against a new target, it is recommended to initially test several antibodies both for sensitivity and specificity toward the target secreted antigen.

## Readout and sorting

The ability to quantify secreted molecules and to sort cells of interest based on these measurements is the final feature that must be considered during the design of single-cell secretion screening technologies. Here, the performance of static microfabricated compartmentalization schemes deviates greatly from droplet-based and lab-on-a-particle-based technologies. Microfabricated wells and compartments are nearly always analyzed via fluorescence microscopy, where the presence of fluorescence reporter molecules can be detected through excitation of the compartment surface. The static nature of microwells combined with the high spatial resolution afforded by fluorescence microscopy enhances the multiplexing capabilities of microwell assays, as secreted analytes can be detected based on combinations of emitted light wavelengths and the localization of capture antibodies in a well, with some reports demonstrating the simultaneous detection of over 40 secreted analytes in a single assay^67^. However, to date, isolation strategies are highly inefficient and typically rely on manual pipetting, which is limited to sorting event rates on the order of 1 cell per minute^68^. Recently, technologies have come to market that enable the parallelized retrieval of cells from compartments using optical^69^ or optoelectronic tweezers^70^, but these novel tools are still prohibitively expensive and not widely available beyond large pharmaceutical companies. In general, microscopy-based analysis and sorting approaches are still limited in the number of single cells that can be analyzed (usually ~1000 per experiment), with the most expensive commercial options containing ~10,000 wells per microwell chip^71^. Screening at this level may be sufficient for some cell therapy discovery efforts but practically cannot be employed for the later stages of cell therapy manufacturing in which millions of cells should be analyzed and sorted.

In contrast, the inherent mobility of microdroplets renders them compatible with increased-throughput continuous sorting modalities. Fluorescence-activated droplet sorting (FADS) is perhaps the most commonly utilized approach for microdroplet isolation^72–74^. In this strategy, an interrogating laser irradiates droplets as they pass through a microfluidic channel, and the resulting fluorescence intensity is recorded. If the intensity of the analyzed droplet is sufficiently above a preselected threshold, an electric field is applied as the droplet passes a downstream junction, and droplets containing cells of interest are diverted into collection reservoirs. While FADS offers significant improvements in both analysis and detection throughputs over microwell array-based sorting, it should be noted that FADS throughputs are typically reported in units of events/second, where an event is any analyzed droplet. While state-of-the-art FADS sorters typically operate at throughputs of ~10^3^ events/second, most analyzed droplets are empty to prevent multicell loading. The actual throughput of cellular events analyzed is typically an order of magnitude less.

Fluorescence activated cell sorting (FACS) is still widely considered to be the gold standard technology for cell sorting, with typical event rates on the order of 10^4^ events/second. Unfortunately, standard flow sorters are incapable of processing water-in-oil emulsions, rendering the translation of microdroplet-based secretion assays difficult. Brower et al. demonstrated the preliminary feasibility of sorting single-cell-containing microdroplets using flow cytometry by forming water-oil-water double emulsions where an outer surfactant-stabilized oil layer preserves droplet stability within the aqueous flow of the cytometer^75,76^; however, the generation of such double emulsions is significantly more complex than that of their single-phase counterparts. Alternative approaches, such as the gelation of aqueous droplets, can provide compatibility with flow cytometry if the gel has secretion capture components^60,77^, but workflows for breaking emulsions and the subsequent recovery of cells or their genetic information from within the microgel are nontrivial^78,79^. Particle-based cell carriers that can isolate cells and capture secretions are ideal candidates for enabling the analysis of single-cell secretions using flow cytometry; however, the microparticle size is often a limiting factor in terms of compatibility with FACS systems. Particles with a range of outer diameters from 30 μm to 85 μm were shown to be compatible with the most common commercial FACS systems^48,52,53^. The protective cavity of the particle also appears to improve the viability of adherent cells within cavities after sorting^48^. Leveraging FACS or magnetic activated cell sorting (MACS) systems that are self-contained, sterile, and qualified for cell production with regulatory bodies might also be the approach best suited for rapid translation into cell therapy manufacturing workflows.

## Example technologies and applications of sorting single cells based on secretions

**Fig. 3:**
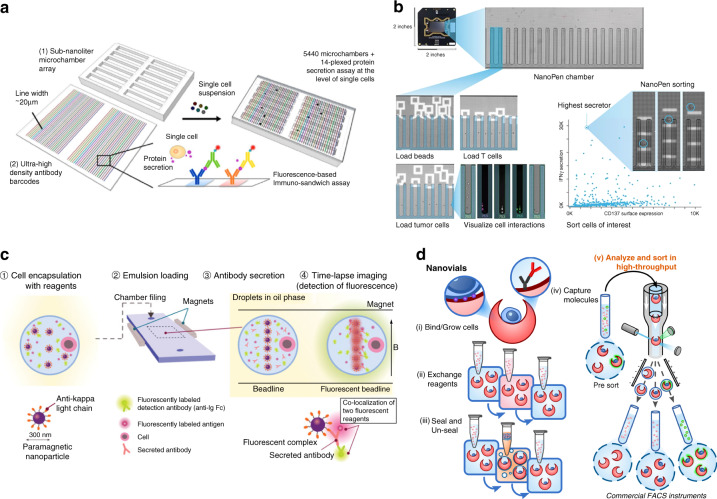
Commercialized Platforms for Single-Cell Secretion Profiling. Several single-cell functional profiling platforms have passed the prototype stage to become full-fledged commercial products. **a** Single-cell barcode chips (commercialized by Isoplexis) are arrays of microfabricated compartments that are useful for the highly multiplexed analysis of secreted products. Compartments are decorated with ordered arrays of antibodies against target proteins, which allows multiplexing based on both the fluorescent signal of reporter molecules and their binding location. **b** Optofluidic nanopen arrays (commercialized by Berkeley Lights) are fabricated on special substrates that are densely loaded with phototransistors that generate dielectrophoretic forces after excitation with high-intensity light. This enables precise manipulation of cells and particles throughout the chip using optoelectronic positioning. The automation and precise control offered by this system enable complex bioassays to be carried out, such as assays that require the coloading of two cells or monitoring of cell growth. **c** In DropMAP, cells are encapsulated into uniformly sized microdroplets alongside capture antibody-functionalized paramagnetic nanoparticles and parked in an incubation chamber. When samples are held in the presence of a magnetic field, the nanoparticles form a visible beadline that yields fluorescent signals in the presence of secreted target proteins. **d** Lab-on-a-particle technologies, such as nanovials, can be modified with various binding moieties to directly facilitate the capture and growth of cells and their secretions. Solutions can be exchanged around cells contained within the nanovials using simple pipetting and centrifugation steps. The emulsification of nanovials in a biocompatible oil and surfactants can limit crosstalk between individual compartments. Particles can be unsealed by breaking the dropicle emulsion. Particles that have captured secreted proteins can be fluorescently labeled and analyzed/sorted with commercial flow cytometry and FACS machines. **a**–**d** Are adapted from references^52,80,147,148^, respectively.

In recent years, several integrated technologies have emerged to analyze and sort cells based on their secretions by leveraging the principles discussed in the concept overview section above. We highlight some of these tools that have successfully transitioned or are transitioning from prototype systems into commercial products. We also discuss the applications of these technologies and how they might be adapted to functionally define or discover cell therapy products. We categorize these technologies based on whether they use microchambers, droplets, or lab-on-a-particle approaches and highlight some of the key performance metrics and features. Figure 3 shows notable examples of each technology.

## Microchambers

Several microchamber-based technologies have been commercialized for analyzing and sorting cells based on secretions, including single-cell barcode chips (SCBCs), optofluidic nanopens, and microcapillary arrays. These techniques have advantages in terms of information density per cell but often lack sorting functionality or require specialized instruments for sorting. Single-cell barcode chips (SCBCs) use concentrated arrangements of microfabricated compartments (typically <1 nL volume) with spatially barcoded capture sites for highly multiplexed single-cell analysis based on secretions (Fig. 3). The defining feature is the DNA-barcoded patterning technology used to spatially localize capture antibodies along the compartment surface^55^. Prior to running an assay, each distinct capture antibody used in a multiplexed assay is localized onto a single vertical bar along the floor of each chamber in the array. This spatial localization allows the identification of secreted proteins based on both the fluorescence emission spectra and the physical location of the reported antibody targeting a specific secreted product. First-generation microchamber arrays were reported in the work of Ma et al.^29^, in which each array was manufactured from polydimethylsiloxane bound to antibody-coated glass substrates, and pneumatic valves were used to seal off each compartment after cell loading. Since this initial prototype, newer SCBC designs have been reported to increase the number of cells (1000 cells/chip)^80^ and number of analytes per cell (40+ simultaneous targets)^68^ that can be analyzed in each assay. Because cells remain in microchambers and no sorting is performed, the technology is limited to the terminal analysis of cell therapy products but not the selection of functional cells in a mixed population.

SCBCs have been commercialized by Isoplexis based on first-generation microchamber technologies^69^. The platform (Isolight) has been used to screen a wide array of cell types, including T cells^81^, macrophages^82^, NK cells^83^, and tumor cells^84,85^, for a multitude of applications. Highlighting the importance of secretions in cell therapy function, Isoplexis has developed a unique metric called the polyfunctional strength index (PSI), which consolidates information on the number of different secreted proteins and the relative amount of each secreted protein into a single value representing the functional activity of an individual screened cell^86^. For CAR-T-cell applications, the PSI metric correlates well with clinical activity. In one analysis, Rossi et al. showed that the presence of larger fractions of highly polyfunctional cells in preinfusion anti-CD19 CAR-T-cell products was predictive of complete response to treatment in patients suffering from aggressive refractory non-Hodgkin lymphoma^87^. Continued validation of such results across large patient populations will prove significant for screening production batches and identifying subsets of patients likely to respond to treatment or to experience significant side effects from therapy, guiding clinical decision-making. Future work employing new technologies to sort and enrich high-PSI cell populations at earlier stages of manufacturing could also be tested to determine whether it would be possible to generate a larger fraction of production batches that lead to optimal clinical responses.

Secretion profiling can also be used in the discovery phase of CAR-T-cell development. In another study, researchers successfully utilized 32-plex Isoplexis cytokine secretion data to identify variations in potency for different CAR constructs, improving the fundamental understanding of the mechanisms behind disease relapse in a phase 1 clinical trial of B-cell acute lymphoblastic leukemia^88^. While limited in scope to the comparison of only two unique CAR constructs, this work alludes to the potential of single-cell functional screening in providing more thorough assessments of CAR function from early-stage in vitro screens. Taken together, the depth of screening offered by SCBC technologies enables researchers to describe variations in cellular functional phenotypes at resolutions that are unattainable with most technologies. Similar chip designs have also been utilized for the multiplexed detection of exosome products^89^, and future design iterations may offer the capability to simultaneously detect novel intracellular biomarkers alongside cytokines. However, it should be noted that the limited number of chambers of the SCBC assay limits its use to relatively common cell populations, and sorting has not been demonstrated, preventing the screening of a random library of CAR constructs.

Sorting within microchamber arrays can be achieved with optofluidic technologies. Optofluidic nanopen arrays are highly ordered arrangements of microfabricated compartments in which cell transport into or out of the compartments is controlled by optically controlled dielectrophoretic forces. The geometry of nanopens generally varies based on design, but each structure consists of deep chambers ranging from a few hundred picoliters to nanoliters in volume, featuring a small opening on one end to facilitate cell loading and nutrient exchange. Uniquely, however, the microfabricated features are patterned onto specialized substrates containing numerous phototransistors that generate dielectrophoretic forces upon excitation with high-intensity light^90^. A benefit of this workflow is that the directed nature of loading is not governed by typical Poisson statistics, permitting higher proportions of chambers on each chip to be loaded with cells. Beads that contain affinity reagents to capture secreted products and other detection reagents can be flowed over the nanopen arrays to perform single-cell secretion assays. Based on the functional output of an individual cell, each cell can be repositioned and isolated from the nanopen array chip for further analysis.

The continued optimization of optofluidic nanopen arrays has led to the creation of an industrial-scale platform, Beacon^®^, developed by Berkeley Lights^70^ (Fig. 3). While this technology is still limited in its cellular analysis throughput due to the footprint constraints on the microfabricated chips (~10,000 pens/chip), the ability to sort cells based on functional outputs provides distinct advantages for preparative sorting in cell therapy workflows for manufacturing and discovery instead of endpoint measurements. Cells, multiple cells, or cells and affinity capture beads can all be simultaneously localized within nanopens, permitting the assessment of increasingly complex biological phenomena such as interactions between immune cells and cells expressing target antigens. In addition, the on-chip expansion of individual cells into clonal populations is permitted^71^, and subsets of expanded populations can be collected into individual wells simultaneously for the concurrent downstream assessment of genotype and phenotype^72,91^. It is not difficult to envision how extensions of preliminary studies reported in the literature can be used to identify beneficial genetic variations in therapeutic cells on the basis of the results of multiplexed cytokine secretion or killing assays. However, rare cell subsets, such as antigen-specific antibody secreting cells or polyfunctional T cells, often comprise <1% of a given population^87,92^. Thus, screening these cell types with SCBC or nanopen technologies often requires preenrichment with either fluorescence or magnetically activated cell sorting (FACS and MACS), a laborious process.

In another variation of microfabricated chambers, cells can also be captured into highly ordered arrays of microcapillaries for subsequent analysis and sorting^93^ using pulsed lasers. Microcapillary arrays are easily loaded through capillary action when the open ends of each chamber come into contact with prediluted cell mixtures. Liquid volumes in each chamber are maintained through surface tension, which allows the suspension of microcapillary arrays over standard microscope setups and direct imaging of settled cells with minimal focusing adjustments. Because of the suspended state, this format was not demonstrated to be compatible with adherent cells. Microcapillary arrays can contain over 1 million individual chambers, but cell loading is dictated by Poisson statistics, meaning that to limit the number of capillaries containing multiple cells, most capillaries will be empty. The technique, first reported by the Cochran lab, demonstrated that laser-induced cavitation can be used to expel the volumes held in each chamber, allowing the recovery of specific cells in capillary volumes on demand^93^ in a workflow the authors call µSCALE (microcapillary single-cell analysis and laser extraction). Automated imaging and laser-assisted recovery were used to screen for antibody secretion and binding, as well as enzyme and fluorescent protein variants produced by mutated libraries of yeast and *E. coli*^94^. Since then, the technology has been commercialized by xCella Biosciences, where it was marketed as the xPloration™ screening platform. Recently, xCella was acquired by Ligand Pharmaceuticals and is now part of their OmniAb^®^ technology suite for the production of humanized antibody products. µSCALE technology is also being used in the cell therapy field by Orca Bio to select functional populations of hematopoietic and other stem cells. By individually selecting cells, Orca Bio proposes to manufacture an optimal cell mixture for reconstituting a patient’s immune system after ablation, potentially preventing graft vs. host disease. Using similar technology, Aridis Pharmaceuticals uses microarrays, called APEX, to enable the screening and single-cell cloning of antibody-secreting B cells^95^.

## Droplet microfluidics

Droplet microfluidics enables the formation and manipulation of many discrete liquid nanoliter- and sub-nanoliter-scale droplets in an immiscible continuous phase^57,73,96^ and has been used for functional assays, such as single-cell secretion assays, by incorporating solid phase beads as affinity capture sites. Unlike other approaches in which solid walls compartmentalize liquids and the number of compartments is limited by physical space in a two-dimensional area, aqueous droplets are isolated in a surrounding oil phase. They can be generated and sorted continuously at a frequency of thousands per second. Therefore, this approach provides unique speed and scalability when applied to single-cell research. The disadvantages include that after sorting, cells must be released from the emulsion state, leading to cell loss, and that analysis of adherent cells in droplets is limited without the presence of a solid surface for cells to adhere. We highlight some of the droplet technologies used for single-cell functional screens that could be applied to cell therapy applications.

DropMAP is a microfluidic droplet-based platform that was first reported in 2017 when Eyer et al. sought to develop a more quantitative and high-throughput technology to screen antibody production from B cells^36^ (Fig. 3). The DropMAP platform has two components. First, a microfluidic droplet generator is used to encapsulate single cells into uniformly sized microdroplets alongside fluorescently labeled detection antibodies and paramagnetic nanoparticles functionalized with capture antibodies. Subsequently, the formed droplets are held in a monolayer within a secondary collection reservoir and incubated to allow the accumulation of secretions from the captured cells. The detection of secreted molecules proceeds in an analogous manner to most other bead-based secretion assays (see detection concept section above), with the notable exception that the paramagnetic capture nanoparticles used in this assay form micron-scale aggregates, termed beadlines, after the application of a magnetic field. This is a critical feature, as the aggregation of capture beads will localize the fluorescence signal in the presence of a high-affinity antigen, improving the signal over the background without washing steps. The use of a large number of microscale beads also overcomes the challenge faced by other techniques with a double Poisson distribution for loading one bead and one cell.

During assays, the collection reservoir is analyzed over time via standard fluorescence microscopy, and the change in fluorescence intensity of each beadline is tracked to monitor the secretion rate and binding affinity of antibodies from ~20,000 individual cells^61^. The detection of secreted molecules occurs rapidly, with some studies reporting the identification of actively secreting cells in <30 min; however, viable cells can also be maintained within compartments for up to 12 h, allowing the observation of changes in secretion kinetics rather than the simple assessment of total protein production as in traditional endpoint assays. This observation mode, without sorting, could be applied to cell therapy product quality control, as for SCBCs, where cytokines or other secreted products of cellular therapeutics are the metrics of potency.

While initial applications of DropMAP technology focused on developing novel insights into the humoral immune response of B cells to vaccines or infections^97,98^, the platform and its reported use cases have expanded with the incorporation of droplet sorting^61^. DropMAP has also been made compatible with on-chip dielectrophoretic sorting and next-generation sequencing (CelliGO™), reportedly enabling the analysis of up to 1 million cells/experiment^99,100^. This technology has been used commercially by HiFiBio. Additionally, the platform enabled the screening of cytokine secretions from T cells after chemical stimulation as well as from primary monocytes isolated from both healthy and septic patients^61^. In combination with droplet sorting, this approach could be applied to enrich functional subsets of nonadherent therapeutic cells (e.g., T cells, B cells, or NK cells) to potentially improve the derived therapeutics made from these more functional base cells.

Other droplet microfluidic technologies are being used to characterize and sort based on functional interactions between cells. For example, HiFiBio demonstrated the screening of bispecific antibodies that link Her2 on a target cell and CD3 on immortalized T cells (Jurkats), leading to activation^101^. They then sorted droplets with activated Jurkat cells to isolate the target cells, which were also engineered as part of a library to produce functional bispecific constructs. This allows sequencing based on function and the screening of a library of constructs based on function. Similar approaches could be applied to screen CARs, T-cell receptors (TCRs), or other constructs being used for the recognition of target cells in the body by a therapeutic cell. In addition to the use of solid-phase beads or target cells, FRET can be used to detect affinity interactions for cell secretions in the solution phase of the droplet containing a secreting cell^102^. This approach, called Cyto-Mine and commercialized by Sphere Fluidics, can be used to screen more than 100,000 cells per experiment and individually dispense clonal colonies for downstream analysis or potential regrowth^35,103^. Another intriguing secretion profiling assay makes use of bispecific antibodies to capture cytokines directly onto the surface of secreting cells, rendering them compatible with traditional immunostaining and droplet microfluidics workflows^104,105^. Unfortunately, these tools are standardly used to interrogate cells in bulk solution, leaving them susceptible to crosstalk between neighboring cells and to loss of signal from cytokines that diffuse away from the local microenvironment. However, a recent study demonstrated the assay in partitioned aqueous volumes, which yielded detectable secretion signals in as little as a few hours while simultaneously increasing assay accuracy and improving the signal intensity on actively secreting cell clones compared to when performing the assay in bulk solution^58^. Although previous reports demonstrate using the technology for cell line development, the ability to screen hundreds of thousands of cells and sort individual cells based on secretory function could be applied to create clonal lines for cell therapies, assuming that clonal populations maintain the same phenotype after many population doublings.

## Lab-on-a-particle systems

Polymeric microparticles can be used as compartments for functional assays: adhering single cells, optionally partitioning them into aqueous compartments called dropicles, capturing secretions on the particles, and finally analyzing and isolating desirable cells based on functional secreted signals through standard flow cytometry^48,52^ (Fig. 3). Notably, while particle fabrication requires microfluidic technology, the fully formed particle samples are stable and can be distributed for use with standard laboratory equipment. This capability substantially facilitates the adoption of this technology compared to microwell or microdroplet technologies, which require specialized instruments. Particles are formed in sizes ranging from 35 µm to 105 µm in diameter, and each contains a single uniformly sized cavity (20–60 µm in diameter) that can be loaded with cells. Cells are attached to particles through a number of approaches, including the targeting of cell surface markers with antibodies or the incorporation of ECM proteins or ECM-derived peptides on the particle. After adhesion, emulsions can be formed through simple pipetting-induced mechanical shear on aqueous particle suspensions in the presence of an oil phase. Once partitioned, cell-loaded particles can be incubated to accumulate secreted products on the particle surface. Finally, the emulsions are destabilized, and the particles can be recovered, stained with fluorescently labeled reporter antibodies or antigens, and sorted with standard FACS. Alternatively, the enclosed shape of the cavity by itself leads to the localization of secretions and local binding that reduces crosstalk to neighboring particles^48^.

**Fig. 4:**
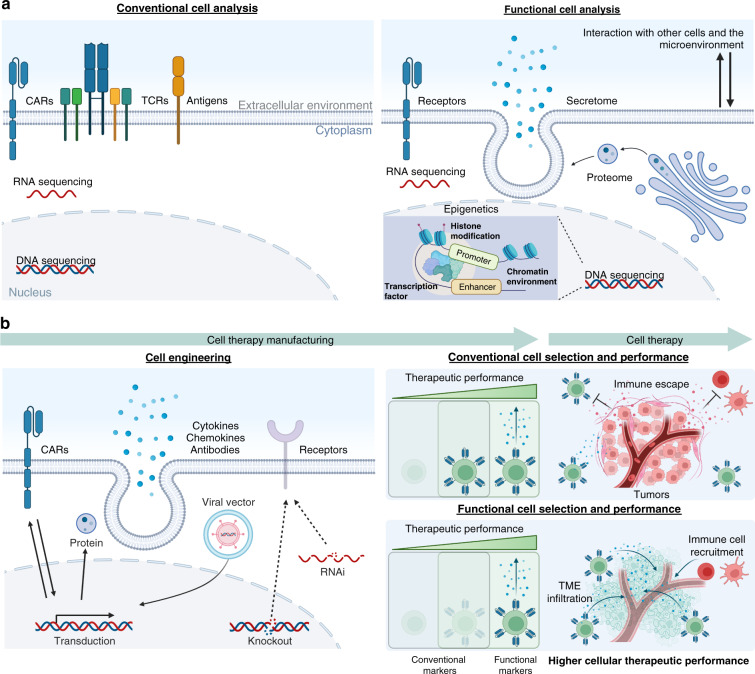
Future cell therapy discovery and manufacturing processes. **a** Single-cell functional profiling technologies can move beyond conventional genotype and surface marker-based analysis. Improved cell therapies will require the direct characterization of cellular function. Unlike conventional sorting systems that rely only on surface marker information, function-based sorting approaches (including those based on secreted products and interactions with other cells or environmental cues) can be helpful in preparing optimal starting cell populations for manufacturing and assessing and purifying cells to improve product quality. **b** Future cell therapies may use functional selection to (1) discover the optimal CAR, TCR or other key engineered functionality and (2) purify the heterogeneous populations of transduced cells for a final high potency formulation after engineering. These selected populations are characterized by functionality and expected to show higher therapeutic performance than conventional cell therapies. For simplicity, a schematic diagram focusing on CAR-T-cell therapies is shown.

The lab-on-a-particle workflow is highly generalizable and has been used successfully with a wide variety of adherent (Chinese hamster ovary (CHO) cells, human embryonic kidney cells, mesenchymal stem cells (MSCs)) and suspension (B cells, T cells, hybridomas, macrophages) cell types for single-cell protein production assays across numerous commercially available flow sorters (BD FACSAria™ II, BDFACSAria™ III, Sony SH800, On-Chip Sort, Union Biometrica Biosorter)^52^. Initial work by de Rutte and coworkers demonstrated the ability to screen and sort highly productive antibody-secreting CHO cell clones as well as antigen-specific antibody secreting hybridomas and murine B cells for antibody discovery applications at overall rates of ~1000 events/second and cell sorting event rates of 100–300 cells/second. Uniquely, in this workflow, cells are encapsulated only for the time necessary to accumulate a detectable amount of secretion signal: several minutes to hours depending on the cell type. For the remainder of the assay, cells are maintained in fresh medium with free exchange of nutrients and waste products. The cavity of the particles has also been shown to protect cells from damage during FACS droplet-in-air sorting, yielding improved viability for adherent CHO cells^48^. These aspects can be particularly beneficial for assays in cell therapy manufacturing workflows, in which sorted populations of cells are used to seed new cultures. One trade-off of the platform is that it requires hands-on time from the end user, as assays are not automated with a complex instrument. However, given the simplicity of the assay workflows and reliance on standard laboratory equipment, it is foreseeable that automated workflows could be performed with robotic liquid handling systems. The open source nature of the platform yields extreme assay customizability depending on user needs. Furthermore, there is no need to preenrich the cell populations of rare cells, as functional readouts can be sorted directly using standard FACS. While the initial work highlighted the feasibility of working directly with primary antibody-secreting cells, a natural extension is evaluation and sorting based on cytokine production from T cells, NK cells or other immune cells for cell therapies. Initial applications span from the discovery of new CAR or TCR constructs to preparing cell populations with optimal functions for downstream manufacturing steps (Fig. 4)^106^. In addition, this platform is the first reported droplet-based screening technology that is fully compatible with adherent cell types and may provide a unique tool to screen understudied cell types, such as MSCs, at the single-cell level to develop more defined functional stem cell therapies. Lab-on-a-particle technology is being made widely available by Partillion Bioscience, which should allow a rapid expansion of applications for this customizable platform based on researcher needs.

## Future perspective: unmet translational needs

While it is true that cellular therapeutics have completely revolutionized the way modern medicine approaches the treatment of systemic and chronic illnesses, the field as a whole is still in its infancy. Cell therapies used to treat disorders work remarkably well for only a small subset of patients and a few diseases^107^. In addition, the personalized nature of modern cell therapies coupled with the difficult manufacturing protocols utilized to generate clinical grade products imparts an astronomically high price tag to the use of cell therapies^108^, such that broad clinical use is currently unfeasible. To address these challenges, we need single-cell functional analysis and sorting technologies beyond conventional genotype- and surface receptor-based analysis. To design improved cell therapies, it will be imperative to elucidate fundamental design rules linking functional cells to their epigenetic and genetic constructs. Function-based sorting approaches can also aid in preparing optimal starting cell populations for manufacturing, assessing and purifying cells to improve product quality^10,109,110^ (Fig. 4). The currently developed single-cell functional screening technologies offer an avenue to accomplish these goals, significantly augmenting the engineering toolkit for cell therapy design and manufacturing. However, significant advances still need to be made to render such tools compatible with the population size scales and levels of resolution needed to be useful. Below, we briefly discuss several unmet needs in the design and manufacturing of cellular therapeutics based on stem cells and immune cells and give thoughts on how improvements in single-cell functional screening technologies may accelerate the realization of robust cell therapies for all (Table 1).

**Table 1:** Current unmet needs and future directions for single-cell functional screening technologies.

Limitation of current analysis	Emerging solutions
• There is a lack of linkage between genotypes and phenotypes. • The presence of CARs in the membrane is not sufficient to know the potency of the cell, taking into account the potential for T-cell exhaustion, metabolic dysfunction, or signaling abnormalities. • Current technology does not allow high-throughput measurement and sorting of cells based on crucial factors that determine the performance of a cell therapy, such as the secretome and proteome.	• Direct characterization and sorting based on cell functions (secretome, proteome) and interaction with other cells and the microenvironment (cell–cell communication, cell-killing, application of force, deposition of ECM) • Functional potency testing of a cell therapy product in vitro prior to use in a patient. • Potential to analyze and sort based on the intracellular proteome and transcriptome while maintaining cell viability.

## Functional screening of stem cells for improving potency

Recent methodological developments have revealed that the underlying benefits of stem cell transplantation might relate not only to the replacement of affected cells at the site of injury but also to paracrine modulatory effects of the transplanted cells^111,112^. Accordingly, a current focus is on the development of technologies for stem cell secretion-based screening^113,114^. Stem cells produce proangiogenic factors, including bFGF, VEGF, and stromal cell-derived factor-1, which is also called chemokine (C-X-C motif) ligand 12 (CXCL12)^115^. In addition, various studies have identified angiogenic factors and their presence in the secretomes from different stem cell sources^111,116–118^. Furthermore, a variety of stem cells have been extensively investigated for their capacity to treat CNS diseases through neurotrophic growth factors such as nerve growth factor, brain-derived neurotrophic factor, ciliary neurotrophic factor, and glial cell line-derived neurotrophic factor^119–122^. However, generally, these secreted products that drive therapeutic potency are not assayed when identifying starting cells for a therapeutic batch, and even potency assays to qualify a batch may not include a robust analysis of secretions. For example, MSCs are the most intensively investigated stem cell type in clinical trials, but the majority of trials fail to reach endpoints, potentially owing to heterogeneity in potency among batches when trials expand to larger numbers of patients. More than 1000 registered clinical trials have explored MSCs for nearly every clinical application, including neurodegenerative and cardiac disorders, GvHD, COVID-19, and cancer. However, most clinical MSC therapies have not met primary efficacy endpoints in clinical trials^123,124^.

Given this context, regulators have issued multiple guidance documents for pharmaceutical companies in the cell therapy field in recent years to improve potency and safety assays, which should start at the earliest stages of product development^8,125–131^. This trend is growing year by year, and many pharmaceutical companies have been forced to delay their development plans following feedback from the FDA due to various issues, particularly involving manufacturing and the quality control tests to assess each cell batch. Consistency is more challenging with cell therapies, which are highly engineered products that can involve a patient’s cells (Fig. 1), than with pharmaceuticals or even the antibody drugs that are now commonplace across the industry. In addition, as many of the cell therapies now in clinical testing are among the first of their type developed, it is not always clear from the beginning what characteristics or attributes are most important for reliably assessing clinical effects.

There are substantial unmet needs to establish new quality control criteria because stem cells are more than a collection of conventional minimal criteria, such as morphology, surface markers, and genetic information. Therefore, we need new cell-quality assessment systems that can ensure the maintenance of the phenotypic and functional properties necessary for treatment rather than relying on conventional genetic and surface marker-based assays. However, most currently available techniques, such as flow cytometry and single-cell sequencing, cannot analyze and sort out cells of interest from a heterogeneous bulk cell population based on their functional properties. Therefore, a new generation of single-cell functional screening technologies is needed to establish robust stem cell-based therapeutic products. Next-generation high-throughput functional cell screening platforms, as discussed herein, can enable the selection of specific clones of stem cells secreting more beneficial secreted factors from bulk cell populations^48,52^. Such features can contribute to the improved potency and reproducibility of clinical products, especially with autologous sources of cells, increase the success rates of clinical trials, and help academic research identify unique functional cell populations for further translational research.

## Improving immune cell therapy product design and quality

In a typical T-cell immunotherapy workflow, cells are withdrawn from a patient, genetically engineered to target tumor cells, expanded ex vivo, and finally reinjected into the patient to systematically search for and destroy the target malignant cells. This personalized therapeutic strategy is helpful in preventing immune rejection, as a patient’s own immune system is used to treat the disease. However, this also means that variations in the basal potency of a patient’s immune cells may fundamentally affect the final efficacy of the drug product^132,133^. In general, it is understood that the main correlates of successful treatments are high levels of in vivo expansion by therapeutic cells after infusion, long-term persistence of infused cells within the body, and high levels of proinflammatory cytokine production after stimulation with cognate tumor antigen; however, it is currently not easy to predict which starting cell populations will excel in each of these areas^4,134^. Several reports have alluded to certain non-terminally differentiated T-cell phenotypes, such as stem-like memory T cells (Tscm) and central memory T cells (Tcm), as potential drivers of the tumor immune response^135^, but sufficient numbers of these cells are not always available in preinfusion products to have appreciable effects. In addition, factors such as patient treatment history and the in vivo disease microenvironment may predispose T cells to enter a metabolically exhausted state with low activity, often resulting in inadequately functional products for the patients most in need.

Single-cell functional screening technologies may be able to directly assist in the selection of highly functional starting cell populations that can improve T-cell product quality. In the near term, longitudinal studies evaluating T-cell surface marker expression and cytokine production and their correlation to patient disease status would provide significant depth to our understanding of functional markers of response. Enriched populations of functionally active cell subsets may be present at the time of treatment or may expand selectively after delivery into the patient to drive responses but may remain unnoticed without functional analysis. As cell therapy is still an early-stage intervention, with costly manufacturing and potential toxicity, improved a priori prediction of patient response would be incredibly beneficial in clinical decision-making regarding who should or should not receive this type of treatment. Additionally, recent reports have begun to indicate that the process of terminal T-cell differentiation and exhaustion may be reversible, for example, through epigenetic remodeling induced by targeted gene disruption^11^, enhanced production of pertinent transcription factors^136^, or small-molecule interventions^137,138^. Functional screens again may directly assess the efficacy of such treatments through evaluation and enrichment of cell subsets that respond most effectively and identification of their transcriptional and epigenetic state. Based on the results of such studies, it may be feasible to implement strategies to broadly recover the functional activity of T cells directly during product manufacturing and select populations of highly secreting and persistent cells as a seed for manufacturing a therapeutic dose with improved potency. Functional screening in immune cell therapies can also be used in the discovery of new therapeutic genetic modifications, such as the use of synthetic CARs, to retarget immune cells. Chimeric antigen receptors (CARs) are designed by pairing target antigen-specific single chain variable fragments (scFvs) with components of the native intracellular T-cell receptor signaling apparatus. While simple in principle, the modular design of CARs can become quite complex, with factors such as the affinity of the scFv, the length of the extracellular hinge region, the structure of the transmembrane region, and the identities of the intracellular costimulatory domains each having a significant impact on the final CAR activity^139^. At present, it is not possible to reliably predict how a specific CAR design will function without laborious empirical testing^140^, and while large libraries (10^6^ constructs) of CARs can be generated and transferred into immune cells simultaneously, only a small fraction of the total library can be analyzed^141,142^. The functional screening technologies described herein may be able to address the screening bottleneck of synthetic CAR molecules by translating functional CAR assessment into a high-throughput screening process that can analyze entire CAR libraries using functional cell sorting technologies. For example, individual clones demonstrating enhanced cytokine production upon exposure to target antigens could be recovered and sequenced to identify constructs of interest. This type of assay may also prove useful for the analysis of unwanted phenomena such as tonic (constitutive or chronic) signaling from CAR constructs in the absence of a target antigen, providing a route to select CAR designs that will result in rapid cell exhaustion. Beyond T cells, this screening and CAR discovery approach can be applied to engineered TCRs and across a number of immune cell types that are being evaluated as therapeutics, including regulatory T cells, γδ T cells, natural killer cells and macrophages^134,143–145^.

## Future perspective: bridging functional analyses with genotypic information

Finally, as a general comment, the ability to pair datasets across multiomics levels of cellular behavior will be a game-changing feature for further refinement of cell therapy products. An abundance of novel single-cell molecular analysis tools have been developed over the past decade, making it possible to analyze a wide array of molecular dimensions at unprecedented resolution. Techniques such as scRNA-seq have been widely adopted due to the production of robust experimental protocols and a growing consensus surrounding the computational approaches for quality control and data analysis. Since single-cell analysis technologies have become widely available, the amount of transcriptomic data has increased exponentially, but recent studies have revealed that transcriptomic data alone cannot accurately predict cellular therapeutic outcomes. For example, mRNA levels and corresponding protein levels are generally not tightly correlated in single cells (Pearson correlation coefficient between −0.12 and 0.40)^146^. Historically, many functional screening techniques, such as ELISPOT or intracellular cytokine staining, have been utilized as endpoint assays, and cellular function could not be paired with gene expression, epigenetic, or sequence information. However, this is not a necessary assay limitation, and single-cell functional analysis technologies are beginning to bridge this gap. A holistic understanding of cell behavior coupled to genetic and epigenetic underpinnings could be used to further engineer cell therapies for improved potency, stability of phenotype, and reduction in toxicity. This can lead ultimately to a new generation of living therapeutics that are well understood and can be controlled with further refinement, extending the benefits to a larger number of patients.
